# Comparative Analysis of Laboratory-Made and Industrial-Made Sewage Sludge Ash: Implications for Effective Management Strategy Development

**DOI:** 10.3390/toxics12050344

**Published:** 2024-05-08

**Authors:** Bartłomiej Michał Cieślik, Oskar Ronda, Elżbieta Grządka, Jolanta Orzeł, Justyna Płotka-Wasylka

**Affiliations:** 1 Department of Analytical Chemistry, Faculty of Chemistry, Gdańsk University of Technology, Gabriela Narutowicza 11/12 Str., 80-233 Gdańsk, Poland; oskar.ronda@pg.edu.pl (O.R.); juswasyl@pg.edu.pl (J.P.-W.); 2 Institute of Chemical Sciences, Faculty of Chemistry, Maria Curie-Sklodowska University, M. Curie-Skłodow-9 ska Sq 3, 20-031 Lublin, Poland; elzbieta.grzadka@mail.umcs.pl (E.G.); jolanta.orzel@mail.umcs.pl (J.O.)

**Keywords:** sewage sludge ash, fluidized bed, solid waste, waste management, surface analysis, toxic elements

## Abstract

In the pursuit of environmentally and economically sustainable sewage sludge ash (SSA) management methods, researchers often employ laboratory-made SSA (L-SSA) as a substitute for industrial-made SSA (I-SSA) produced in fluidized bed furnaces. To check whether L-SSA is a material that imitates I-SSA well, the fractionation of metals whose presence is a significant problem during SSA management was performed. In addition, the grain distribution, specific surface area, and textural properties of the tested materials were examined. Differences in total Pb and Hg content and mobility of Cu, Ni, Mn, and Zn were observed between I-SSA and L-SSA. Larger particle sizes of L-SSA compared to I-SSA were confirmed, while comparable textural properties and specific surface area of both types of materials were maintained. Based on the results, it was concluded that L-SSA is chemically different compared to I-SSA, and that L-SSA should not be used as a reference in research focused on the design of SSA management methods. Moreover, fractionation of metals was performed in disposed fluidized beds (FBs), which are diverted to non-hazardous waste landfills without prior analysis. It has been proven that studied metals are present in FBs as abundantly as in SSA, while Cu, Mn, and Ni may show higher mobility than in I-SSA.

## 1. Introduction

Nowadays, both raw sewage sludge and its processing products are beginning to be perceived not only as waste but also as a valuable material [1,2]. One of the modern methods of post-fermentation sludge management, thanks to which it is possible to both recover energy and obtain potentially useful raw material, is thermal utilization by combustion in fluidized bed furnaces. As a result of sewage sludge incineration in a fluidized bed furnace, three fractions of waste are usually obtained. Sewage sludge ash (SSA) is a fraction constituting a mineral residue after the combustion process, most often collected in the first set of filters after thermal utilization of SS. Another fraction is solid residues after exhaust gas treatment, also called air pollution control (APC) residues, consisting of very fine solid particles and gases bound as a result of the adsorption process on sorbents in the second flue gas dedusting system. The last piece of waste is a disposed fluidized bed (FB), which constitutes a contaminated silica fraction. The aforementioned fluidized bed is replaced in the furnaces as part of annual technical and service shutdowns. 

Disposed fluidized bed is a minority fraction of waste that arises from the need to periodically replace the fluidized bed in the furnace (usually once a year). It is classified in the EU as non-hazardous waste [3] and, for this and other reasons, it is usually omitted in works dealing with waste generated as a result of thermal utilization of sewage sludge in fluidized bed furnaces. However, during its operation in the furnace, the fluidized bed is in contact with thousands of tons of incinerated sewage sludge, containing, among other things, significant amounts of heavy metals. In view of this fact, there is a suspicion that disposed-of fluidized beds may constitute waste with a high content of this group of hazardous substances, and thus this fact should be taken into account when looking for effective and economically justified ways to manage this waste fraction.

SSA obtained as a result of sewage sludge incineration constitutes the vast majority of the total mass of waste generated during this process (usually 60–90%). Ash, due to its increasing production (growing with the growing popularity of sewage sludge incineration technologies), as well as its physicochemical characteristics, is the object of interest of both the scientific community and the industry in the context of searching for management methods. It is characterized by a high content of phosphorus—several to over a dozen percent by weight (in some cases even up to 20% by mass) [4,5]. Apart from the aforementioned elements, sewage sludge ash mainly contains compounds of silicon, aluminum, iron, sulfur, sodium, calcium, and magnesium, and to a lesser extent compounds of other metals, including highly toxic compounds of cadmium, lead, or mercury [5,6,7,8,9,10]. The share of mobile forms of some metals, such as Cu or Zn, may be significant in the context of the environmental risk associated with the storage or management of this type of waste, which is necessarily associated with the introduction of the aforementioned fractions into the environment [11,12]. These materials can be used as a relatively safe means of soil reclamation [13]. Another method of management could be the use of ash as a phosphate fertilizer or for the recovery of phosphorus compounds from them, from which it is possible to produce synthetic phosphate fertilizers [4,6,14,15]. In the literature, one can also find the idea of creating concretes and cements with good mechanical properties, containing SSA [16,17,18,19] and lightweight aggregates [20,21,22]. Potentially, ash could also be used as a sorbent for metals from polluted waters [23,24,25,26] and, due to the high content of metals in their composition, as sorbents for some gases (e.g., hydrogen sulfide) [27,28,29]. 

Some works concerning the development of ash management methods and focusing on discovering their chemical characteristics may, however, be burdened with a serious methodological error regarding the use of ash obtained by the combustion of sewage sludge in laboratory furnaces (laboratory-made SSA—L-SSA) as a research material, instead of using ash from industrial installations, usually based on technology using fluidized bed furnaces (industrial-origin SSA—I-SSA) [23,27,28,30,31,32,33,34,35,36]. The combustion conditions in both types of furnaces are radically different and can result in obtaining a product with different physicochemical properties. In fluidized bed furnaces, combustion is carried out at a temperature of 850 °C, under conditions of intensive mixing and grinding of sewage sludge. Therefore, the time necessary to completely burn a unit portion of the material is only a few seconds. In laboratory furnaces, due to the lack of mixing and grinding of the material, the combustion of one portion of sewage sludge (ranging from a few to several hundred grams) lasts from one to several hours, depending on the weight of the sludge being burned. Considering the differences in time of the combustion process, there is a high probability that the ash after sewage sludge incineration in laboratory conditions may have a lower content of volatile substances at the combustion temperature [37], as well as different speciation characteristics of heavy metals (which may change during prolonged exposure to high temperature of the material) [38]. In addition, L-SSA may have different granulation characteristics and different surface properties due to the lack of mixing, grinding, and abrasion of the burnt material.

In the case of works concerning the use of ash in soil reclamation, as an additive to building materials and as a phosphate fertilizer, the key is not only the content but also the mobility of heavy metals, which may vary depending on the conditions of sewage sludge incineration, as has already been discussed. The speciation characteristics of metals contained in the tested material are also important when considering the use of ash as a gas sorbent in chemical adsorption. In this aspect, the surface characteristics and grain size distribution of the potential sorbent also seem to be of key importance. The specifics of the grain size distribution of ash also play a role in the efficiency of such processes as phosphorus recovery, and can also have a significant impact on the quality and properties of the obtained cement and aggregates containing this material. The discussion on the possible effects of using L-SSA as a test material for research on the characteristics and management of SSA in the examples of selected papers is presented in Table 1.

The novelty of this work is the study of the differences in the content and mobility of selected metals, specific surface area, and ash grain distribution based on the sewage sludge combustion technology used (laboratory furnace, fluidized bed furnace). An additional aim of the work was to determine the reliability of the test results for ash obtained in laboratory conditions as a research material. The characterization of disposed fluidized beds, which is omitted in the scientific literature, was considered crucial in the context of determining the holistic impact of SS thermal utilization processes on the environment, and to the best of the authors’ knowledge, no studies on the chemical characteristics and potential ways of managing contaminated fluidized beds have been conducted so far. The presented research concept constitutes an element of scientific novelty and emphasizes the issue of selecting appropriate samples as research material in order to carry out the process of waste management development.

## 2. Materials and Methods

### 2.1. Test Material

The research material consisted of two types of SSA produced in sewage sludge treatment plants with incineration facilities. They were ash obtained during the combustion of sewage sludge with the use of technologies based on thermal treatment in fluidized bed furnaces and ash obtained during the incineration of SS from the same wastewater treatment plant in muffled furnaces. Samples of I-SSA came from three sewage treatment plants: the Gdańsk Wschód Sewage Treatment Plant, the Group Sewage Treatment Plant in Łódź (GOŚ Łódź), and the Group Sewage Treatment Plant Dębogórze in Gdynia (GOŚ Dębogórze). The total number of collected ash samples was 15 samples of L-SSA and 16 samples of I-SSA. In addition to ash, fluidized bed samples, used for the process of thermal utilization of sewage sludge in fluidized bed furnaces for a minimum of 300 days, were also collected (overall 5 samples). The details of the technology used in different installations may vary slightly; however, the incineration stage was carried out at the same temperature (850 °C) in every considered installation. 

To obtain L-SSA, samples of dried sewage sludge that were collected from the same plants during the same time as I-SSA were placed in porcelain crucibles and incinerated at 850 °C for 2 to 3 h (depending on the degree of sludge fragmentation). This time was defined as the minimum combustion time enabling complete combustion of the organic matter (manifested as the disappearance of the black color of the sludge), which made it possible to obtain ash with characteristics similar to those of ash after combustion in fluidized bed furnaces (carbon content below 5%). The L-SSA samples prepared in this way were considered ready for comparative analyses with I-SSA. Although the presented research takes the form of a case study, the conclusions drawn from the research (repeated in the case of every three installations considered) should be universal.

### 2.2. Fractionation of Metals

Operational speciation (fractionation) of heavy metals and aluminum was carried out using the sequential extraction technique according to the modified European Community Bureau of Reference (BCR) sequential extraction method (Figure S1 in Supplementary Materials). The elements selected for the analysis are the ones most commonly analyzed by researchers in the field of SSA management. However, it is important to notice that the content and speciation of other potentially toxic elements such as metalloids can also be a vital part of the comparative analysis of I-SSA and L-SSA, which should be the subject of future research The proposed extraction method allows for the separation of four fractions of heavy metals—carbonate and ion exchange (F1), those associated with Fe and Mn oxides (F2), sulfide and organic (F3), and the residual fraction (F4). Fractions F1 and F2 are considered highly mobile in the environment. F3 is a fraction referred to as temporarily immobile, and F4 is completely immobile [39]. Two-times-smaller amounts of individual reagents were used in stages 1–3 compared to the standard BCR sequential extraction procedure [40]. 

The applied procedure is shown in Figure S1. Such a modification was aimed at lowering the limit of detection and determination of elements by limiting the dilution of the sample and economizing the chemical reagents used. A comparison of the results of analyses obtained using standard and twice-smaller amounts of reagents is included in Table S1 (Supplementary Materials). It was proven that in the case of the tested materials, the use of smaller amounts of reagents can result in the determination of lower F1 fractions of some metals; however, this does not have a significant impact on the possibility of drawing conclusions about differences in the chemical characteristics of the tested materials. The essence of the presented research is the comparative analysis of the leaching potential of metals under the same extraction conditions for all tested types of materials. It should be noted that the BCR method is not the only method for separating metal fractions, and the use of other procedures may lead to significantly different analysis results [41]. Therefore, it was decided to use a single, consistent extraction method throughout the entirety of the research. All reagents used in the sequential extraction were high-purity reagents intended for metal trace analysis.

The extracts obtained as a result of the described procedure were analyzed using microwave-induced plasma optical emission spectrometry (MIP-OES). The contents of zinc, chromium, aluminum, cadmium, manganese, copper, nickel, lead, and iron were determined in the extracts. The MP-AES 4210 device from Agilent was used in the presented research.

Table S2 (Supplementary Materials) presents selected validation parameters of the applied analytical methods based on the MIP-OES technique. If the determination of a given element was carried out using different wavelengths, the arithmetic mean was taken as the result of the analysis. Wavelengths were selected from the most intense emission band characteristic for a given element in such a way as to avoid possible interference between a given analyte and other elements present in the sample at significant concentration levels.

### 2.3. Determining Total Mercury Content

As part of the presented research, the total mercury content in samples was determined using atomic absorption spectrometry with cold vapor atomization (CV-AAS). It was decided not to fractionate mercury due to its high volatility (possible significant analyte losses during sample heating), low concentrations of this metal in the tested materials, and the fact that mercury compounds in any form pose a significant environmental hazard.

Approximately 100 mg of ash (with an accuracy of 0.1 mg) were weighed into the thermally treated boats compatible with the Shimadzu MA-3000 mercury analyzer device (Shimadzu, Kyoto, Japan). The validation parameters of the method used are presented in Table S3 (Supplementary Materials).

### 2.4. Surface Analysis

In order to determine the surface properties of the tested materials and find the most important differences between I-SSA and L-SSA, a number of physicochemical analyses were performed, such as the measurement of particle diameter and specific surface area, as well as porosity characterization.

The dynamic light scattering (DLS) method was used to measure the average particle size of I-SSA. The obtained results are presented as average particle sizes, and the polydispersity, which is a measure of the differentiation of particle sizes in the tested system, is given.

Due to the much larger particle sizes of L-SSA, a sieve analysis was used to study the grain size distribution in this type of material. Twenty sieves with mesh sizes ranging from 0.05 mm to 7.00 mm were used. Each fraction separated by sieving the material through a specific sieve (from the smallest to the largest) was weighed on an analytical balance. The results are presented as mass percentages of fractions of given sizes.

Specific surface areas and pore characteristics of the tested samples were determined using the low-temperature nitrogen adsorption–desorption technique using the BET method (ASAP 2420, Micrometrics, Norcross, GA, USA). This technique consists of determining the nitrogen adsorption isotherm at the temperature of 77.0–77.5 K in accordance with the BET model. Pore volumes and pore diameters were analyzed by low-temperature N2 adsorption using the BJH method [42]. This method is based on the Kelvin equation with a modification involving multilayer adsorption [43]. Micropore volumes were calculated with the t-plot method using the Harkins–Jur equation [44] based on the above N2 adsorption data.

## 3. Results and Discussion

Due to the wide scope of the tests, in order to facilitate the analysis of the results, this chapter has been divided into subchapters in which the elements of the physicochemical characteristics of the tested materials are discussed separately.

### 3.1. Metal Fractionation Results

This subchapter discusses the results of the determination of individual fractions of selected heavy metals in ash. The results are presented in the form of box plots. Error bars indicate the minimum and maximum value of a given parameter characterizing the samples. The median of the results is marked as a horizontal line inside the colored rectangle marking the first and third quartiles. The arithmetic mean of the results is marked with the symbol “X”.

#### 3.1.1. Zinc

**Figure 1:**
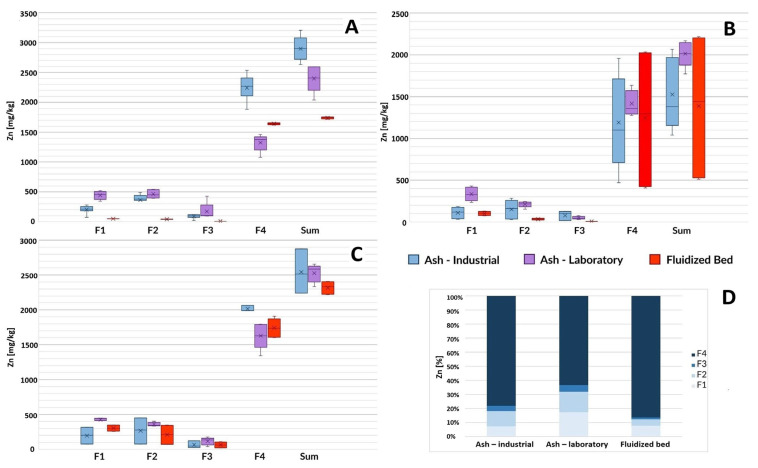
Zinc content in ash samples from three wastewater treatment plants based on the sludge incineration method (laboratory furnace or industrial installation) and in disposed fluidized beds. F1—ion exchange and carbonate fraction, F2—fraction associated with Mn and Fe oxides (reducible), F3—organic and sulfide fraction, F4—residual fraction, Sum—the total content of the element. (**A**) GOŚ Łódź, (**B**) Gdańsk Wschód, (**C**) GOŚ Dębogórze, (**D**) percentage fraction by material type (average of all treatment plants).

The total zinc content in the tested materials was similar regardless of the origin of the sample and the method of production (in a fluidized bed furnace or in a laboratory) and ranged from 1400 to 2800 mg/kg (average values) (Figure 1). This content of zinc should be considered high, although fractionation showed the dominance of immobile forms of this element (residual fraction). Ash obtained by incineration of sewage sludge in laboratory conditions was generally characterized by a higher share of highly mobile forms (fractions F1 and F2). This difference is particularly important in the case of F1, where the average concentrations of zinc in the mentioned form in L-SSA ranged from 335 mg/kg to 451 mg/kg, compared to the zinc content in the range of 108 mg/kg to 203 mg/kg in the discussed fraction w in I-SSA. Zinc contained in fluidized beds may occur in less mobile forms than in ash, although the total content of this element in fluidized beds and ash was similar.

#### 3.1.2. Manganese

**Figure 2:**
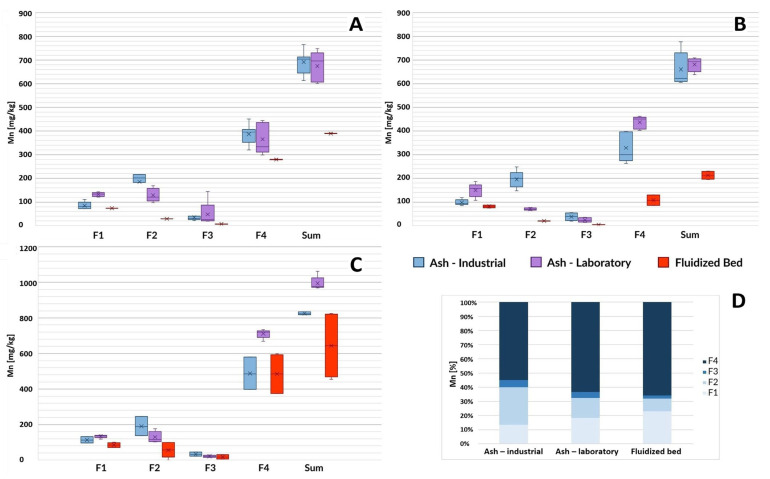
Manganese content in ash samples from three wastewater treatment plants based on the sludge incineration method (laboratory furnace or industrial installation) and in disposed fluidized beds. F1—ion exchange and carbonate fraction, F2—fraction associated with Mn and Fe oxides (reducible), F3—organic and sulfide fraction, F4—residual fraction, Sum—the total content of the element. (**A**) GOŚ Łódź, (**B**) Gdańsk Wschód, (**C**) GOŚ Dębogórze, (**D**) percentage fraction by material type (average of all treatment plants).

As in the case of zinc, the tested ash from three sewage treatment plants had a similar total manganese content (Figure 2). Differences in the chemical composition of I-SSA and L-SSA could be identified only after performing operational speciation with the use of sequential extraction. Laboratory ash was characterized by, on average, 1.2–1.6 times higher content of ion-exchange species (F1) and, on average, 1.4–2.7 times lower content of reducible fraction (F2), which in this case should probably be associated with a lower content of manganese (III) oxide and manganese (IV) oxide.

Under the conditions of the second stage of extraction, these compounds may undergo reduction, and then manganese enters the solution in the form of Mn^2+^ ions [45]. However, this hypothesis requires confirmation using screening speciation, which allows for the unambiguous determination of the chemical species mentioned. The tested samples of fluidized beds were characterized by a lower total manganese content in relation to ash (on average 1.8, 3.1, and 1.3 times lower for samples from Gdańsk Wschód, GOŚ Łódź, and GOŚ Dębogórze, respectively), with a relatively high share of ion exchange fraction (F1) and a very low content of F2 and F3 fractions compared to ash obtained on an industrial scale (Figure 2D). The percentage of mobile F1 and F2 manganese fractions in laboratory ash (18% and 14%, respectively) was more similar to the characteristics of fluidized beds (23% and 9%, respectively) than I-SSA (14% and 26%, respectively).

#### 3.1.3. Copper

**Figure 3:**
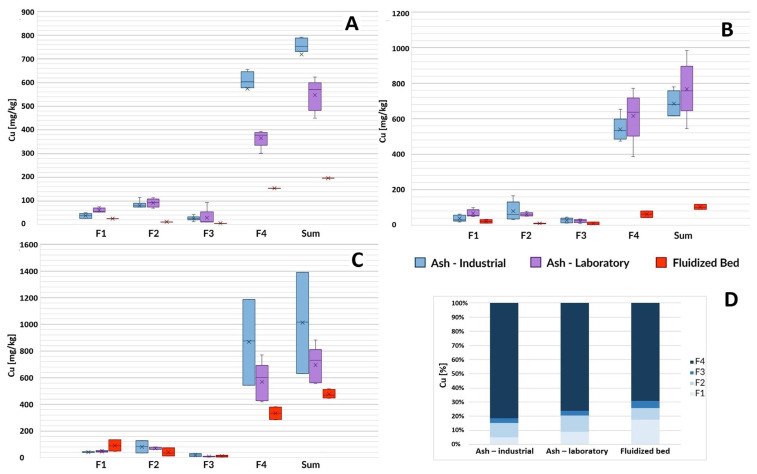
Copper content in ash samples from three wastewater treatment plants based on the sludge incineration method (laboratory furnace or industrial installation) and in disposed-of fluidized beds. F1—ion exchange and carbonate fraction, F2—fraction associated with Mn and Fe oxides (reducible), F3—organic and sulfide fraction, F4—residual fraction, Sum—the total content of the element. (**A**) GOŚ Łódź, (**B**) Gdańsk Wschód, (**C**) GOŚ Dębogórze, (**D**) percentage fraction by material type (average of all treatment plants).

No reproducible differences were observed between laboratory and industrial ash in the total content of copper (different distribution of results depending on the sewage treatment plant, ranging from 546 to 1013 mg/kg (average values) for both types of ash). As in the case of zinc and manganese, the dominant copper fraction was the residual fraction (F4) (Figure 3). L-SSA was characterized by a slightly higher (9% of the total Cu) content of ion exchange and carbonate fractions (F1) compared to I-SSA (5% of the total Cu). The tested samples of fluidized beds contained significantly (on average, 2.1–6.3 times) less copper compared to I-SSA, and the mentioned material was generally characterized by a higher relative content of F1 fraction (18% of the total Cu) and a lower relative content of F2 fraction (8% of the total Cu) compared to the fly ash from fluidized bed furnaces (5% of F1 and 10% of F2 fractions).

#### 3.1.4. Nickel

**Figure 4:**
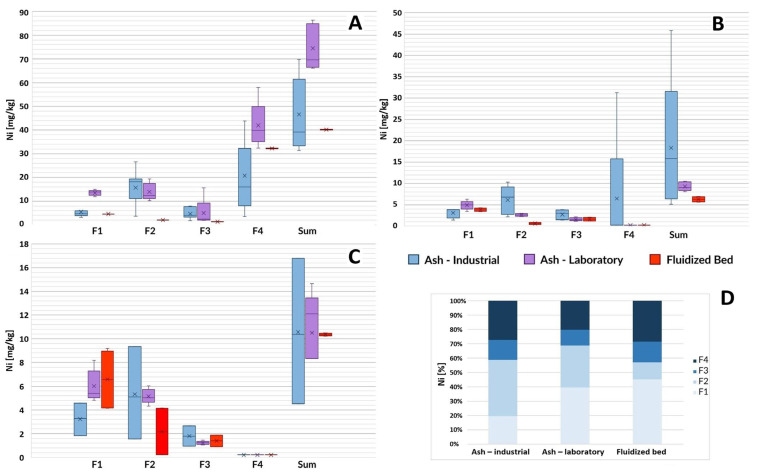
Nickel content in ash samples from three wastewater treatment plants based on the sludge incineration method (laboratory furnace or industrial installation) and in disposed-of fluidized beds. F1—ion exchange and carbonate fraction, F2—fraction associated with Mn and Fe oxides (reducible), F3—organic and sulfide fraction, F4—residual fraction, Sum—the total content of the element. (**A**) GOŚ Łódź, (**B**) Gdańsk Wschód, (**C**) GOŚ Dębogórze, (**D**) percentage fraction by material type (average of all treatment plants).

The most diverse analysis results the tested ash, depending on the place of origin of the sewage sludge, were obtained for nickel (Figure 4). The average total content of this element in laboratory samples ranged from 9.3 mg/kg in samples from the Gdańsk Wschód sewage treatment plant to 74.5 mg/kg in samples from GOŚ Łódź. The total nickel content in samples from fluidized bed furnaces ranged from 10.6 mg/kg in samples from GOŚ Dębogórze to 48.3 mg/kg in samples from GOŚ Łódź. Nickel is the only one among the determined elements for which the dominance of the residual fraction (F4) in both ash and fluidized beds cannot be unequivocally stated. Based on the conducted research, it is not possible to explain the nature of the observed phenomenon, which suggests the need for further research. The content of individual fractions of nickel in the tested materials was varied and specific for individual sewage treatment plants. The only consistent observation was the 1.6–2.5 times higher content of ion exchange and carbonate fractions (F1) in L-SSA compared to I-SSA (depending on the sewage sludge incineration plant). It is important to emphasize the particularly high relative share of the F1 fraction in the disposed fluidized beds (45%), which was similar to the average content of this fraction in L-SSA (39%) and significantly higher compared to I-SSA (19%).

#### 3.1.5. Lead

**Figure 5:**
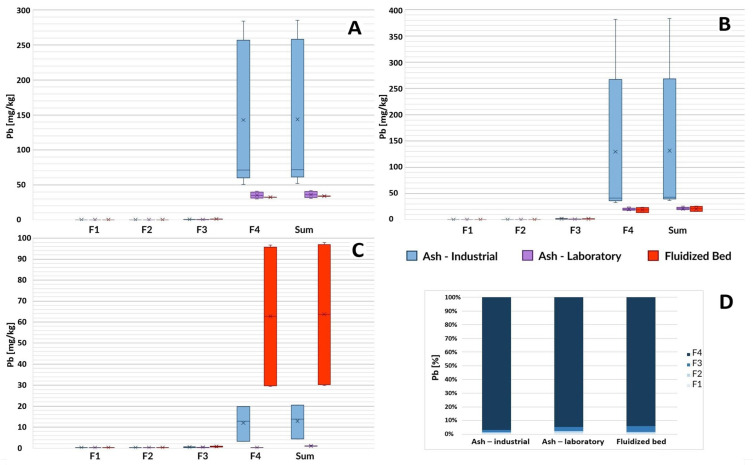
Lead content in ash samples from three wastewater treatment plants based on the sludge incineration method (laboratory furnace or industrial installation) and in disposed fluidized beds. F1—ion exchange and carbonate fraction, F2—fraction associated with Mn and Fe oxides (reducible), F3—organic and sulfide fraction, F4—residual fraction, Sum—the total content of the element. (**A**) GOŚ Łódź, (**B**) Gdańsk Wschód, (**C**) GOŚ Dębogórze, (**D**) percentage fraction by material type (average of all treatment plants).

Based on the analysis, it was shown that ash after the incineration of sewage sludge can contain various amounts of lead (Figure 5). In the case of samples from the Gdańsk Wschód and GOŚ Łódź sewage sludge thermal treatment plants, the content of this toxic element was typically within the range of 35–270 mg/kg (Figure 5A,B), while in the case of GOŚ Dębogórze it was only in the range of 3–20 mg/kg. This element occurred in the tested materials almost exclusively (>97% in I-SSA, <94% in L-SSA, and <93% in FB) in immobile forms (residual fraction—F4), although L-SSA was characterized by a 4.2–14 times lower lead content than its counterpart obtained by burning sewage sludge in fluidized bed furnaces (I-SSA). Lead compounds, due to their weak metallic nature, are often characterized by relatively low melting and boiling points. Probably during the long process of burning sewage sludge in laboratory furnaces, some lead compounds evaporated from the sample. The evaporation of lead is probably due to the formation of lead chloride, which is highly volatile under combustion conditions [46]. An alternative theory may be the reduction of lead compounds to metallic lead and its intensive evaporation at the incineration temperature. Testing these hypotheses requires the use of analysis in the form of individual speciation in order to determine the content of specific chemical compounds of lead and to confront their physicochemical properties with the conditions of the combustion process. Intensive evaporation of lead and its compounds during the combustion of sewage sludge in laboratory conditions was confirmed by another team [38]. Disposed fluidized beds from Łódź (Figure 5A) and Gdańsk (Figure 5B) contained significantly less lead than ash taken from the same installations, which is justified analogously to the comparison of L-SSA and I-SSA (longtime of firing the bed in the furnace). However, an anomalously high concentration of lead in fluidized beds was observed in samples from Gdynia (Figure 5C).

#### 3.1.6. Chromium, Aluminum, Cadmium, Iron

The graphical presentation of the results of chromium, aluminum, cadmium, and iron determination is presented in Supplementary Materials, because no statistically significant differences were observed in the content of chromium, aluminum, cadmium, or iron between laboratory and industrial-origin ash (Figures S2–S5). Ash after sewage sludge incineration contained significant amounts of chromium (even over 1000 mg/kg), almost exclusively (>99%) in immobile forms (residual fraction—F4). The content of this element in fractions F1, F2, and F3 was below the limit of quantification (<0.98 mg/kg) in all tested samples of L-SSA and I-SSA. The content of cadmium in the ash was relatively low, usually in the range of 3–10 mg/kg, with a clearly marked predominance of immobile forms (residual fraction—F4), which constituted on average 72%, 65%, and 79% of the total cadmium content in the tested I-SSA, L-SSA, and FB samples, respectively. Aluminum and iron in the ash were present in very high amounts (usually 36–94 g/kg of Fe and 16–62 g/kg of Al) and were some of the main components of the tested materials. The main reason for this is the fact that coagulants based on aluminum and iron salts (so-called PAX and PIX, respectively) are used in the process of chemical wastewater treatment. As a result of chemical reactions in the coagulation process, practically water-insoluble aluminum and iron compounds are formed, which end up in the sewage sludge. These elements occurred in the ash mainly in the F4 fraction, which constituted over 99% of the total content of Al and Fe. Fluidized beds had a similar or slightly lower content of the elements listed in this subchapter, and the relative share of individual fractions was very similar to the distribution characteristic of ash (Figures S2–S5).

#### 3.1.7. Mercury

**Figure 6:**
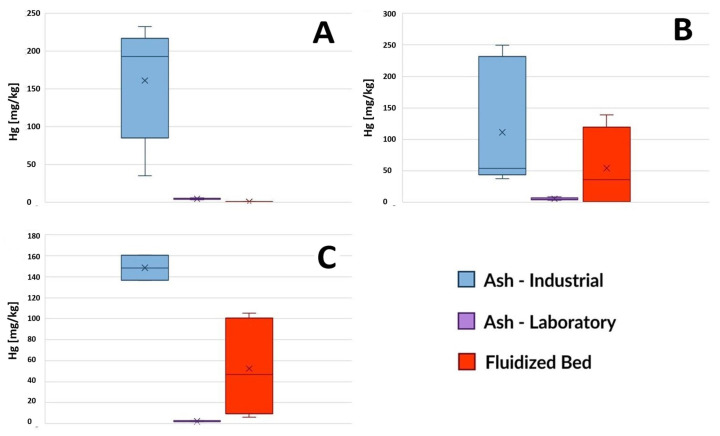
Total mercury content in ash samples from three wastewater treatment plants, based on the method of sludge incineration (laboratory furnace or industrial installation) and in disposed-of fluidized beds. (**A**) GOŚ Łódź, (**B**) Gdańsk Wschód, (**C**) GOŚ Dębogórze.

Ash after sewage sludge incineration was characterized by a low mercury content compared to the content of the other heavy metals tested. The direct reason for this is the ease with which mercury compounds were reduced to metallic mercury, which then quickly evaporated from the sample in the conditions of the incineration process. Trace amounts of mercury (ranging from 1 to 9 µg/kg) were determined in L-SSA that had been in the furnace for 2–3 h (Figure 6). I-SSA had a much higher mercury content (from 35 µg/kg to 250 µg/kg). A possible explanation for this observation is the fact that the sewage sludge incinerated in the fluidized bed furnace stayed there for only a few seconds, after which the ash and vapors were immediately cooled down, which may have enabled secondary adsorption of mercury vapor on the surface of recrystallized material collected in the first set of bag filters, forming I-SSA fraction. The disposed-of fluidized beds were characterized by varying mercury content, although generally lower than that of I-SSA (ranging from 0.4 to 138.4 µg/kg). The dominance of the Hg^0^ speciation form and therefore the intensive vaporization of this element during sewage sludge combustion was confirmed in the fluidized bed furnace [47] and in the laboratory furnace [48] as well. 

### 3.2. Measurement Results of the Textural Properties of the Tested Materials

Based on the analysis of the data contained in Table 2, it can be concluded that laboratory and industrial ash are characterized by similar textural parameters. In most cases, I-SSA had a more developed specific surface area, but the differences were so small that a similar sorption capacity of both types of materials is expected. As one can see, the total pore volumes in the I-SSA materials were usually larger in comparison to the L-SSA ones, but their average pore diameters were similar, which means that the I-SSA materials had bigger pores. There were no significant differences in the micropore volumes of the L-SSA and I-SSA materials. Attention should be paid to the differing values of the specific surface area and the volume of pores and micropores in the ash from GOŚ Dębogórze compared to the materials from the other two sampling places. It is also interesting that in the GOŚ Dębogórze materials, the greatest differences were found between L-SSA and I-SSA. This observation shows that the ash, because of significant differences in specific surface area and pore volumes, may differ in sorption capacity depending on the technological solutions used in the wastewater treatment plant at the stage of its production. Therefore, research on the use of ash after incineration of sewage sludge as a sorbent should be carried out using samples of ash from various sewage treatment plants in order to avoid underestimating or overestimating the typical sorption properties of SSA.

**Figure 7:**
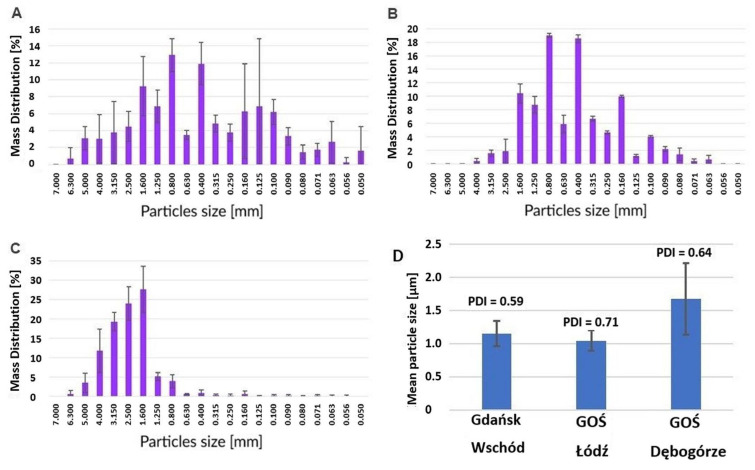
The particle diameter distributions of the tested samples of laboratory-made ash (L-SSA): (**A**) GOŚ Łódź, (**B**) Gdańsk Wschód, (**C**) GOŚ Dębogórze, (**D**) industrial origin (I-SSA).

Figure 7 presents the particle diameter distributions of the tested samples of laboratory ash obtained with the use of sieve analysis. In the case of L-SSA from the sewage treatment plants Gdańsk Wschód and GOŚ Łódź, particles of similar sizes were observed, mostly in the range of 0.315 to 0.800 mm. As in the case of the analysis of textural properties, the characteristics of the samples from GOŚ Dębogórze were different. Here, the dominant particle diameters were in the range of 1.25–4.00 mm. A probable explanation for this phenomenon may be technological differences between the wastewater treatment plants at the stage of the preparation of sludge for incineration (dewatering, drying, granulation). In the case of I-SSA, this parameter may also have been significantly affected by the quality of the fluidized bed (diameter of its particles) and the specifics resulting from the technology of the dust collection system used.

The average particle diameter of I-SSA was about 1 µm, although, as in the case of laboratory samples, the ash from GOŚ Dębogórze was characterized by a larger diameter. The particle size distribution of the tested materials was wide, as evidenced by high polydispersity (PDI) values (0.59–0.71). I-SSA was characterized by particle sizes three orders of magnitude smaller than its counterpart obtained by incinerating sludge in laboratory furnaces. This is due to the extremely intensive grinding processes that occur during the mixing and abrasion of the sludge with the fluidized bed in the fluidized bed furnace. In view of the observations made, it should be clearly stated that the use of L-SSA in research work, in which the size of the material particles plays a significant role, is unreliable. Laboratory ash in no way constitutes a representative research material unless L-SSA is milled to a particle size equivalent to that of I-SSA.

### 3.3. Overall Analysis of Research Results

There were noticeable differences in the mobility of some metals (zinc, copper, manganese, nickel) between I-SSA and L-SSA. The samples obtained in a laboratory were characterized by a higher relative content of the ion exchange fraction (F1) of the mentioned metals. This fraction is critical from the point of view of a possible risk of environmental contamination connected with the secondary use of ash due to its particularly high mobility. This risk may be overestimated if an inference is made on the basis of tests performed using samples obtained on a laboratory scale. The phenomenon of the partial immobilization of metals in ash is probably caused by the formation of spinel structures. These structures are formed at high temperatures during the thermal treatment of materials with high iron content and are responsible for the immobilization of elements such as chromium, copper, and zinc [49]. Differences in the mobility of metals contained in I-SSA and L-SSA may therefore result from differences in the dynamics of the formation of spinel structures depending on the sludge combustion conditions used. However, this hypothesis requires confirmation in further research. In general, I-SSAs are characterized by a relatively low mobility of heavy metals; therefore, despite the high total metal content, the risk associated with their leaching is usually not high. Similar conclusions can be drawn by analyzing the results of previous studies on the chemical characterization of I-SSA carried out by other teams [11,50]. However, when comparing the results obtained by other teams, it should be borne in mind that in the case of using operational speciation of metals using sequential extraction according to the BCR model, there are common problems with the reproducibility of results during inter-laboratory comparisons [51]. On the basis of the data contained in Table S4 (Supplementary Materials), attempts were made to estimate the types of metals in the case of which the process of their leaching from the tested materials may significantly increase their concentration in the soil. The soil environment is most exposed to contamination when ash is used as a fertilizer, as an additive to building materials, or in land reclamation, as well as when it is stored in open landfills. The estimated typical content of heavy metals in soil in Poland was given on the basis of the report [52] for chromium, zinc, cadmium, copper, nickel, manganese, and lead, and on the basis of published literature data [53] for aluminum and iron. The largest content of mobile fractions of metal in ash in relation to the content of that element in the soil was recorded for copper and zinc; therefore, these elements may pose the highest risk of environmental contamination in the case of attempts to manage ash. The risk associated with mobile fractions of cadmium is unspecified, due to the insufficient sensitivity of the method used to determine this element. Some effects of soil exposure to ash can be seen for manganese and nickel. In the case of the remaining metals tested, the risk of soil contamination related to the leaching of the mentioned metals from the ash can be considered marginal.

An important observation is that L-SSA had significantly lower total mercury and lead content compared to I-SSA. This is particularly important when considering the possibility of the direct use of ash as a macronutrient inorganic fertilizer, as a soil amendment, or as a raw material for the production of these products. According to the legislation of the European Union [54], the content of mercury in such products cannot exceed 1 mg/kg, and that of lead, 120 mg/kg (presented as total content). Samples of ash from the Gdańsk Wschód sewage treatment plant were generally characterized by a higher lead content than 120 mg/kg, which means that they could not be used as a macronutrient fertilizer or soil amendment. The use of L-SSA as a research material leads to an opposite, false conclusion. Taking into account the literature data [5,6,11,50], it is concluded that the lead content in ash very often exceeds the maximum allowable content of this metal in the cited legal standard. Therefore, it should be assumed that the use of L-SSA may lead to a complete falsification of reality in the context of the possibility of ash management in the discussed manner.

Based on the analysis of the surface characteristics of the tested ash samples after the thermal treatment of sewage sludge, it can be concluded that laboratory samples and samples from industrial installations will have similar sorption capacities. However, due to the relatively low values of specific surface areas obtained for these samples, the ability of unmodified ash to adsorb impurities should be considered low—definitely lower than that of typically activated carbons.

Many teams postulate the use of ash as a hydrogen sulfide sorbent due to the high content of metals [27,28]. These metals would adsorb hydrogen sulfide by chemical adsorption with the formation of sulfides. It should be considered certain that the efficiency of sulfide formation depends on the speciation forms of metals present in the sorbent. Due to the different chemical nature of L-SSA and I-SSA, proven by fractionation using the BCR sequential extraction technique, it should be considered that the adsorption efficiency of hydrogen sulfide on L-SSA and I-SSA may differ. In connection with the above, it should be emphasized that the results of research conducted in this area using samples of ash produced in a laboratory may be unreliable.

It was proven that fluidized beds contaminated as a result of the sewage sludge incineration process, similar to ash, may have contained significant amounts of metals that are potentially hazardous to the environment, and in the case of some metals (Cu, Ni, Mn) the share of the most mobile ion exchange fraction (F1) was significant and greater than that of ash. An additional, important observation is the fact that the distribution of the relative share of individual fractions of most metals in fluidized beds was more similar to the distribution characteristic of L-SSA than of I-SSA. This may be related to the duration a fluidized bed is in the furnace, which is significantly longer than that of sewage sludge and the ash generated from it (300 days vs. a few seconds). As a result of prolonged exposure of the material to high temperatures, changes in the chemical forms of the metals contained in it may occur, similar to what happens during long-term incineration of sludge in a laboratory furnace. It has already been proven that the duration of sewage sludge incineration can have a significant impact on the relative share of individual fractions of some metals in the residue [38].

## 4. Conclusions

Based on the analysis of the research results presented in this work, it was concluded that ash obtained as a result of the thermal utilization of sewage sludge was characterized by partly different chemical and physical characteristics depending on the incineration conditions (laboratory furnace, fluidized bed furnace). Ash obtained in a laboratory furnace (L-SSA) was characterized by significantly lower total mercury and lead content than its counterpart produced in industrial installations based on fluidized bed furnace technology (I-SSA). Moreover, a higher share of mobile fractions of zinc, manganese, copper, and nickel was noted in L-SSA. On the other hand, there were no significant differences in the values of parameters characterizing the specific surface area of the tested ash based on the production technology, although L-SSA was characterized by a grain diameter at least several hundred times larger than that of I-SSA. Based on the observations made, it can be stated that L-SSA is not a proper equivalent of ash from industrial installations and cannot be considered a representative material for research in which the total content of Pb and Hg; the mobility of Zn, Mn, Cu, and Ni in SSA; or the grain diameter of the material is crucial. Due to the above, the results of some studies on the characteristics and management of ash after the thermal treatment of sewage sludge, conducted with the use of samples of ash produced on a laboratory scale, may be unreliable and should be subject to critical assessment. The authors propose to always use ash coming directly from industrial installations (regardless of the specific research purposes) in order to avoid doubts regarding the reliability of the results obtained due to the research material used. Moreover, greater attention should be paid to the need to take into account the high content of certain potentially environmentally hazardous metals in disposed-of fluidized beds from sewage sludge incineration furnaces when planning the disposal or management of this waste fraction. In some cases, the relative content of mobile metal fractions in fluidized beds may be higher than in ash. The high content of the most mobile fractions of copper and nickel in fluidized beds is particularly disturbing in the context of environmental hazards.

## Figures and Tables

**Table 1:** Discussion of possible effects of using L-SSA as a test material for research on the characteristics and management of SSA in the examples of selected papers.

Aim of the Work	Key Stages of L-SSA Sample Preparation	Potential Errors Founded	Possible Effects on the Characteristics of the Obtained Material	References
Using SSA as an adsorbent of Cu from synthetic wastewater	Combustion at 700 °C for 3 h Milling and screening to separate particles with diameter < 75 nm	The temperature of combustion is lower than that typically used on an industrial scale (850 °C) Milling after combustion, not during the process	The surface properties of the obtained L-SSA can be different compared to that of I-SSA because of no mixing and grinding of the material during the combustion process. The particle size can be still higher than I-SSA from a fluidized bed furnace.	[23]
Using SSA as a sorbent of H_2_S from the gas mixture	Combustion at 900 °C for 2 h No information about milling and screening	The temperature of combustion is lower than typically used on an industrial scale (850 °C) No milling or screening	The higher temperature of combustion and other different conditions of combustion (laboratory furnace vs. fluidized bed furnace) can lead to obtaining different speciation of metals in ash. This can affect the efficiency of the chemical sorption of H_2_S. The surface properties of the obtained L-SSA can be different compared to I-SSA because of no mixing and grinding of the material during the combustion process. The particle size will be significantly higher than that of I-SSA from a fluidized bed furnace.	[27]
Characteristic of the mobility of selected metals in SSA depending on the temperature of the combustion. Assessment of the ecotoxicological aspect.	Combustion at 850 °C, 900 °C, 950 °C, 1000 °C for 60 min at maximum temperature	The time of the combustion can be too short to complete the burning of organic matter. The final content of organic matter was not analyzed. I-SSA The conclusion about the ecotoxicological aspect based on L-SSA	Different conditions of combustion (laboratory furnace vs. fluidized bed furnace) can lead to obtaining different speciation of metals in ash, which can make the conclusion about differences in the chemical characteristics of ash depending on the temperature of the combustion incorrect. Moreover, this can affect the environmental risk.	[30]
Extraction of phosphorus from SSA and heavy metal removal from obtained extracts	Combustion at 850 °C, 4 h Milling (no specific information)	Milling after combustion, not during the process	The particle size can be higher than I-SSA from a fluidized bed furnace, which can affect the efficiency of the extraction process due to the smaller contact surface with the extractant. Different conditions of combustion (laboratory furnace vs. fluidized bed furnace) can lead to obtaining different speciation of phosphorus and metals in ash (more or less difficult to extract).	[32]

**Table 2:** Results of measurements of the textural parameters of ash. The method of presenting the results: mean ± standard deviation.

Sewage Treatment Plant	Type of Sample	BET Surface Area (m^2^/g)	Total Pore Volume (cm^3^/kg)	T-Plot Micropore Volume (cm^3^/kg)	BJH Adsorption Average Pore Diameter (nm)
Gdańsk Wschód	L-SSA	3.66 ± 0.20	15.81 ± 0.22	0.40 ± 0.14	24.6 ± 2.0
	I-SSA	3.70 ± 0.91	20.08 ± 6.05	0.31 ± 0.14	28.0 ± 3.6
GOŚ Łódź	L-SSA	3.12 ± 0.20	13.31 ± 1.59	0.34 ± 0.08	23.0 ± 1.6
	I-SSA	3.58 ± 1.12	13.21 ± 6.02	0.36 ± 0.13	20.0 ± 5.5
GOŚ Dębogórze	L-SSA	0.88 ± 0.20	2.90 ± 0.76	0.17 ± 0.05	26.9 ± 9.3
	I-SSA	1.79 ± 1.00	8.12 ± 4.11	0.20 ± 0.07	27.9 ± 3.6

## Data Availability

Dataset available on request from the authors.

## Supplementary Materials

The following supporting information can be downloaded at: https://www.mdpi.com/article/10.3390/toxics12050344/s1, Figure S1: Diagram of the metal fractionation procedure used; Figure S2: Cadmium content in ash samples from three wastewater treatment plants based on the sludge incineration method (laboratory furnace or industrial installation) and in disposed-of fluidized beds; Figure S3: Iron content in ash samples from three wastewater treatment plants based on the sludge incineration method (laboratory furnace or industrial installation) and in disposed-of fluidized beds; Figure S4: Chromium content in ash samples from three wastewater treatment plants based on the sludge incineration method (laboratory furnace or industrial installation) and in disposed-of fluidized beds; Figure S5: Aluminum content in ash samples from three wastewater treatment plants based on the sludge incineration method (laboratory furnace or industrial installation) and in disposed-of fluidized beds; Table S1: Comparison of metal fractionation results obtained for SSA and FB using standard amounts of reagents for sequential extraction (BCR model) and twice-smaller amounts (modification); Table S2: Presentation of selected validation parameters of the methods used for the determination of heavy metals; Table S3: Validation parameters of the mercury determination method; Table S4: Comparison of the content of mobile fractions of the tested metals in ash from industrial installations and fluidized beds, and the concentration of these metals in Polish soils [39,40].
