# Supplementary material for: Comparative Analysis of Laboratory-Made and Industrial-Made Sewage Sludge Ash: Implications for Effective Management Strategy Development
Source: Toxics. 2024 May 8;12(5):344. doi: 10.3390/toxics12050344 (PMC11125665; doi:10.3390/toxics12050344)
Supplement: Supplementary file 1 [file toxics-12-00344-s001.zip › toxics-2938787-Supplementary materials.pdf]

Table S1. Comparison of metal fractionation results obtained for SSA and FB using standard amounts of reagents for sequential extraction (BCR model) and twice smaller amounts (modification)

Material	Amount of reagents	Fraction	Fe [mg/kg]	Zn [mg/kg]	Cd [mg/kg]	Cu [mg/kg]	Ni [mg/kg]	Mn [mg/kg]	Cr [mg/kg]	Al. [mg/kg]	Pb [mg/kg]
I-SSA	2x smaller amount	F1	< 11	297 ± 19	< 0.83	67.8 ± 1.9	4.93 ± 0.14	110.5 ± 6.7	< 0.30	< 16	< 0.45
I-SSA	2x smaller amount	F2	35.0 ± 2.0	275 ± 18	0.92 ± 0.11	184 ± 11	11.43 ± 0.10	259.0 ± 1.0	< 0.30	< 16	< 0.45
I-SSA	2x smaller amount	F3	< 3.3	20.4 ± 4.1	< 0.83	19.7 ± 1.5	2.74 ± 0.32	29.6 ± 6.1	< 0.98	< 5.0	< 0.45
I-SSA	2x smaller amount	F4	59600 ± 1300	1710 ± 52	4.82 ± 0.11	824 ± 18	< 0.41	365 ± 10	183.0 ± 9.8	50480 ± 950	36.3 ± 1.5
I-SSA	Standard BCR model	F1	< 22	385 ± 36	< 1.7	141.1 ± 7.6	9.28 ± 0.21	210.0 ± 4.0	< 0.60	< 32	< 0.90
I-SSA	Standard BCR model	F2	166 ± 11	410 ± 44	< 1.7	179.3 ± 5.4	10.49 ± 0.34	222.5 ± 7.5	< 0.60	36.8 ± 4.6	< 0.90
I-SSA	Standard BCR model	F3	< 6.6	36.1 ± 6.2	< 1.7	28.3 ± 4.0	2.97 ± 0.23	25.0 ± 3.3	< 2.0	< 32	< 0.90
I-SSA	Standard BCR model	F4	59800 ± 400	1263 ± 26	4.910 ± 0.020	740 ± 26	< 0.82	311.6 ± 4.3	186.3 ± 7.7	50370 ± 470	37.2 ± 1.9
Fluidized bed	2x smaller amount	F1	< 11	130.7 ± 6.4	0.870 ± 0.020	37.4 ± 2.2	4.11 ± 0.43	97.4 ± 4.2	< 0.30	< 16	< 0.45
Fluidized bed	2x smaller amount	F2	81.1 ± 3.3	44.4 ± 3.0	< 0.83	12.0 ± 2.4	< 1.4	19.5 ± 2.6	< 0.30	41.7 ± 4.7	< 0.45
Fluidized bed	2x smaller amount	F3	< 3.3	< 9.7	< 0.83	2.1 ± 1.1	< 1.4	4.2 ± 1.4	< 0.98	< 5.0	< 1.5
Fluidized bed	2x smaller amount	F4	24300 ± 1100	512 ± 83	< 0.83	94.6 ± 2.8	< 0.41	136.8 ± 4.7	114.6 ± 8.5	12780 ± 370	26.6 ± 3.2
Fluidized bed	Standard BCR model	F1	23.4 ± 4.9	158 ± 13	< 1.7	42.4 ± 2.9	4.58 ± 0.53	106.7 ± 4.4	< 0.60	69.4 ± 3.8	< 0.90
Fluidized bed	Standard BCR model	F2	154.5 ± 5.2	33.5 ± 9.7	< 1.7	15.0 ± 2.3	< 2.8	20.4 ± 1.0	< 0.60	178.9 ± 8.3	< 0.90
Fluidized bed	Standard BCR model	F3	< 6.6	< 5.8	< 1.7	3.3 ± 1.2	< 2.8	5.4 ± 1.4	< 2.0	< 10	< 0.90
Fluidized bed	Standard BCR model	F4	24200 ± 900	493 ± 111	< 0.50	88.6 ± 3.9	< 0.82	128.7 ± 7.8	115 ± 8	12550 ± 160	26.6 ± 2.4

The MIP-OES technique is one of the modern spectrometric techniques characterized by high sensitivity to most metals, the possibility of determining many elements during one analysis, and low analysis costs due to the use of pure nitrogen as a plasma source. In this case plasma gaz (nitrogen) can be obtained from the air using a compressor equipped with appropriate molecular sieves. It is a significant compromise between flame atomic absorption spectrometry (F-AAS) and electrothermal atomic absorption spectrometry (ET-AAS), which have many limitations, and the highly expensive inductively coupled plasma - mass spectrometry (ICP-MS) and inductively coupled plasma - optical emission

spectrometry (ICP-OES). The use of the described technique in elemental analysis brings many benefits and is in line with the principles of green analytical chemistry.

Table S2. Presentation of selected validation parameters of the methods used for the determination of heavy metals (MIP-OES technique)

Element	*Wavelength [nm]*	*Calibration curve equation*	*Coefficient of determination (R^2)*	*LOD [mg/kg]*	*LOQ [mg/kg]*
Chromium	425.433	I = 4732.5124 · C + 17.4998	0.9998	0.30	0.98
Zinc	213.857	I = 6427.4757 · C + 1896.0791	0.9986	2.90	9.70
Aluminium	394.401	I = 4096.7266 · C + 10349.0066	0.9992	5.00	16.00
Cadmium	228.802	I = 13278.3979 · C + 14.2457	0.9997	0.25	0.83
Manganese	403.307	I = 16619.2712 · C + 16.9282	0.9999	0.19	0.63
	403.076	I = 25053.0881 · C + 10.1911	0.9999	0.26	0.82
Copper	324.754	I = 74480.8257 · C + 482.1520	0.9997	0.27	0.89
	327.395	I = 44382.9856 · C + 178.4845	0.9999	0.31	1.00
Nickel	352.454	I = 11475.1738 · C – 47.9812	0.9999	0.41	1.40
Lead	405.781	I = 2074.2240 · C – 3.1355	0.9987	0.45	1.50
Iron	371.993	I = 5445.2246 · C – 1165.1406	0.9995	3.30	11.00

Table S3. Validation parameters of the mercury determination method (CV-AAS technique)

Element	*Wavelength [nm]*	*Calibration curve equation*	*Coefficient of determination (R^2)*	*LOD [μg/kg]*	*LOQ [μg/kg]*
Mercury	257.000	A = 0.68045 · C + 0.00089	0.9956	0.05	0.18

Table S4. Comparison of the content of mobile fractions of the tested metals in ashes from industrial installations and fluidized beds, and the concentration of these metals in Polish soils (Chief Inspectorate of Environmental Protection, 2020; Ronda et al., 2022)

Element	*Highly mobile fraction content (F1 + F2) in I-SSA (mean ± standard deviation) [mg/kg]*	*Highly mobile fraction content (F1 + F2) in fluidized bed (mean ± standard deviation) [mg/kg]*	*Typical concentration in soil (Poland) [mg/kg]*

Chromium	< 0.60	< 0.60	12
Zinc	443 ± 231	365 ± 229	62
Aluminium	< 44	< 38	4300
Cadmium	< 1.4	< 0.80	0.65
Manganese	285 ± 42	117 ± 42	379
Copper	119 ± 46	73 ± 68	6.3
Nickel	14.7 ± 8.4	< 6.6	9.7
Lead	< 0.46	< 0.46	29
Iron	< 49	249 ± 119	3600

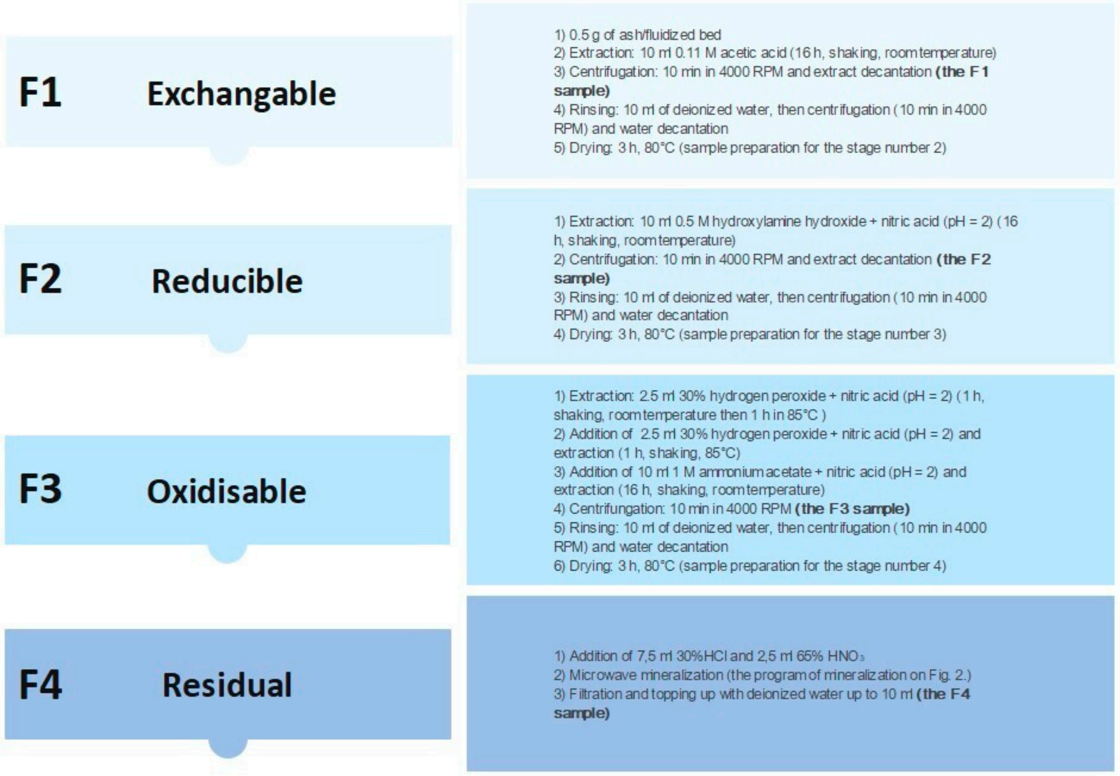

Figure S1. Diagram of the sequential metal extraction procedure used.

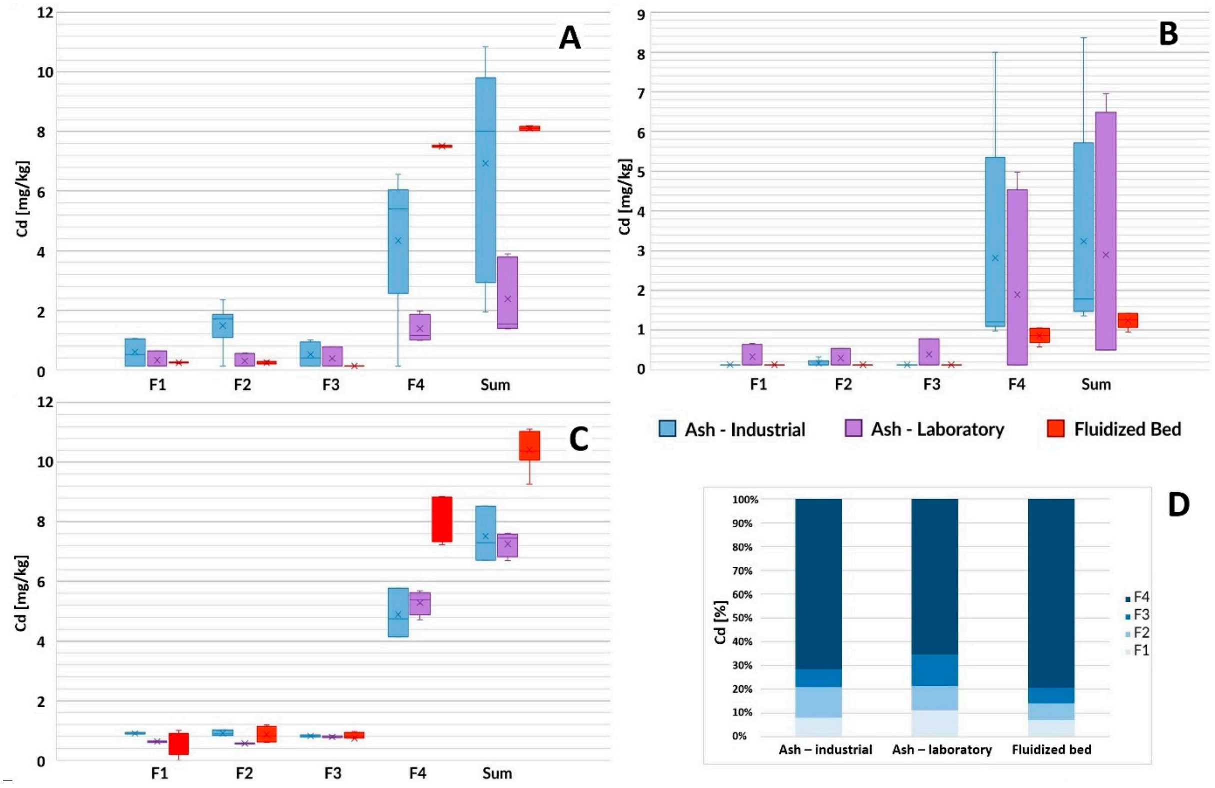

Figure S2. Cadmium content in ash samples from three wastewater treatment plants depending on the method of sludge incineration method (laboratory furnace or industrial installation) and in used fluidized beds. F1 – ion exchange and carbonate fraction. F2 – fraction associated with Mn and Fe oxides (reducible). F3 - organic and sulphide fraction. F4 - residual fraction. Sum – the total content of the element. A – GOŚ Lodz. B - Gdansk East. C – GOŚ Dębogórze. D – percentage fraction by type of material (average of all treatment plants).

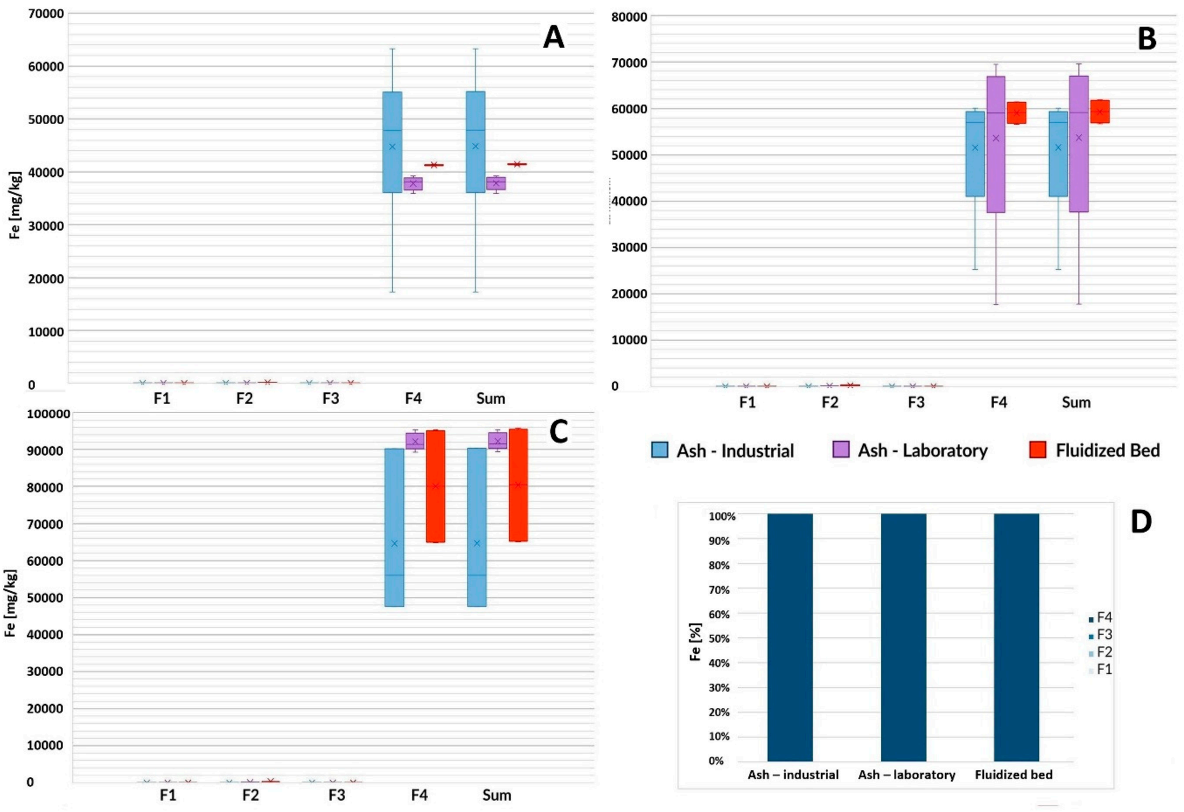

Figure S3. Iron content in ash samples from three wastewater treatment plants depending on the method of sludge incineration method (laboratory furnace or industrial installation) and in used fluidized beds. F1 – ion exchange and carbonate fraction. F2 – fraction associated with Mn and Fe oxides (reducible). F3 - organic and sulphide fraction. F4 - residual fraction. Sum – the total content of the element. A – GOŚ Lodz. B - Gdansk East. C – GOŚ Dębogórze. D – percentage fraction by type of material (average of all treatment plants).

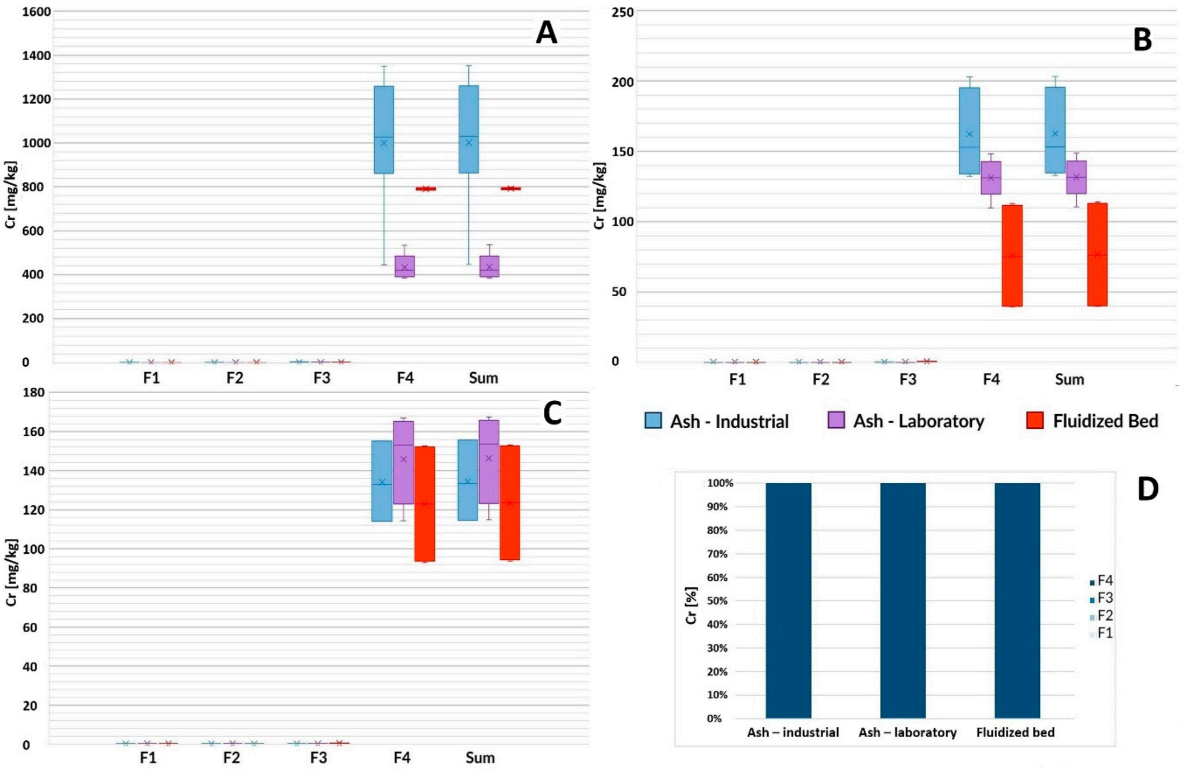

Figure S4. Chromium content in ash samples from three wastewater treatment plants depending on the method of sludge incineration method (laboratory furnace or industrial installation) and in used fluidized beds. F1 – ion exchange and carbonate fraction. F2 – fraction associated with Mn and Fe oxides (reducible). F3 - organic and sulphide fraction. F4 - residual fraction. Sum – the total content of the element. A – GOŚ Lodz. B - Gdansk East. C – GOŚ Dębogórze. D – percentage fraction by type of material (average of all treatment plants).

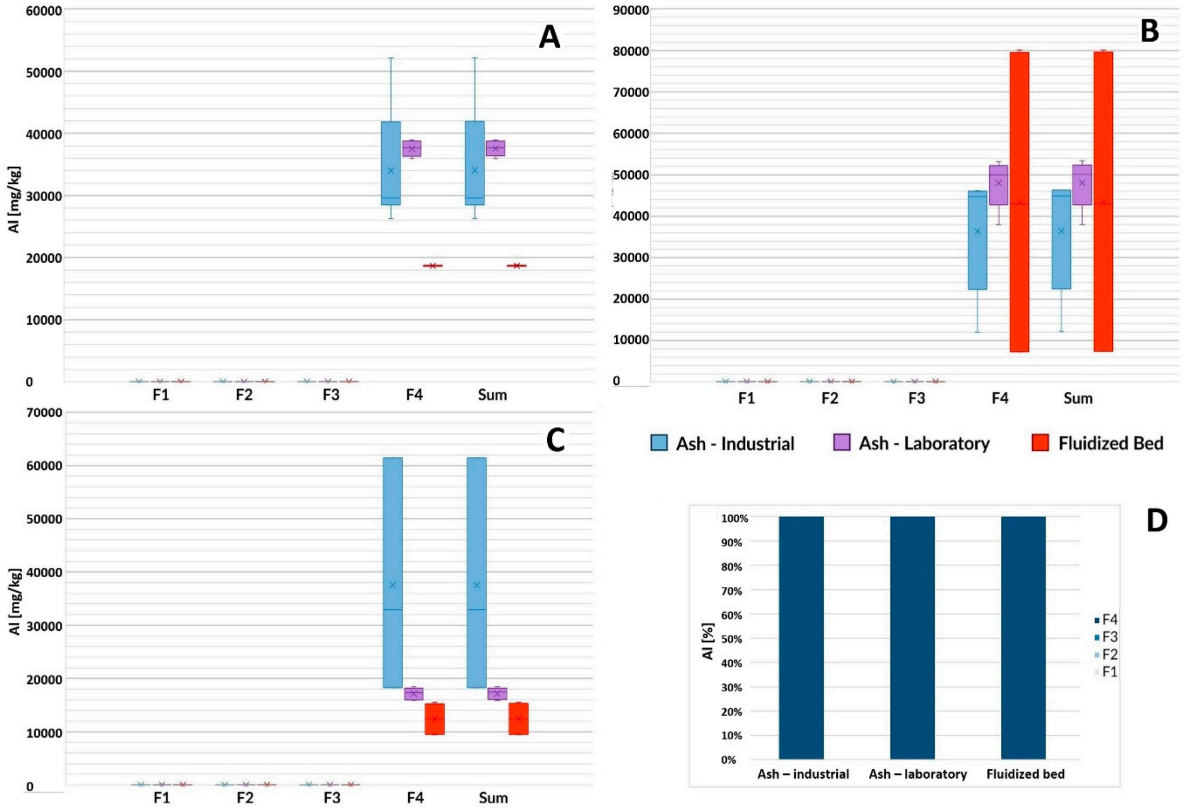

Figure S5. Aluminum content in ash samples from three wastewater treatment plants depending on the method of sludge incineration method (laboratory furnace or industrial installation) and in used fluidized beds. F1 – ion exchange and carbonate fraction. F2 – fraction associated with Mn and Fe oxides (reducible). F3 - organic and sulphide fraction. F4 - residual fraction. Sum – the total content of the element. A – GOŚ Lodz. B - Gdansk East. C – GOŚ Dębogórze. D – percentage fraction by type of material (average of all treatment plants).
